# Shining light on knee osteoarthritis: an overview of vitamin D supplementation studies

**DOI:** 10.3389/fmed.2024.1423360

**Published:** 2025-01-22

**Authors:** Di Zhang, Miaoyu Ye, Yao Xu, Luyu Jiang, Yanmei Hu, Qi Zhang, Xiao Han, Qian Dai, Junhui Qian, Jian Luo, Qiang Yuan

**Affiliations:** ^1^School of Acupuncture and Tuina, Chengdu University of Traditional Chinese Medicine, Chengdu, China; ^2^Department of Tuina, Hospital of Chengdu University of Traditional Chinese Medicine, Chengdu, China

**Keywords:** vitamin D supplements, keen osteoarthritis, overview, systematic reviews, quality assessment

## Abstract

**Background:**

The impact of knee osteoarthritis on individuals’ daily functioning is significant. In recent years, Vitamin D supplements cure osteoarthritis has garnered attention from medical professionals and patients due to its simplicity and portability. Several systematic reviews (SRs) and meta-analyses (MAs) have examined the efficacy of vitamin D supplementation for knee osteoarthritis, yet there is variability in their methodology and quality.

**Objective:**

To search, gather, and analyze data on the characteristics and quantitative results of SR/MA in patients with KOA treated with Vitamin D supplementation, and objectively evaluate the efficacy of supplements. Then, provides clinical evidence and recommendations the clinical use of vitamin D supplementation.

**Methods:**

Two individuals reviewed and collected data from four databases until October 2023. AMSTAR-2, ROBIS, PRISMA 2020, and GRADE tools were used to evaluate the methodological quality, bias risk, reporting quality, and evidence strength of all SR/MA. Additionally, we applied the corrected covered area (CCA) method to measure overlap in randomized controlled trials (RCTs) cited among the SR/MA.

**Results:**

3 SRs and 6 MAs were included in the analysis: 3 studies were low quality by AMSTAR-2, and 6 studies were very low quality. According to ROBIS, 6 studies were high-risk and 3 were low-risk. In PRISMA 2020 reporting quality, most studies showed deficiencies in comprehensive literature search strategy, reasons for literature exclusion, data preprocessing for meta-analysis, exploration of reasons for heterogeneity, sensitivity analysis, publication bias, and disclosure of funding and conflicts of interest. Grading the quality of evidence in GRADE consisted of 5 items of moderate quality, 14 items of low quality, and 10 items of very low quality. Bias risk and imprecision were the main factors for downgrading. The calculation of RCT overlap between SR/MA using CCA showed a high degree of overlap.

**Conclusion:**

Vitamin D supplementation may show potential efficacy in ameliorating symptoms of KOA. The evidence indicates that Vitamin D supplements for knee osteoarthritis can improve patients’ Total WOMAC scores and synovial fluid volume in the joints. Nevertheless, due to the generally low quality of current studies, future research should prioritize improving the quality of primary studies to establish the efficacy of vitamin D supplementation for KOA with more robust scientific evidence.

**Systematic review registration:**

The protocol of this overview was registered in the International Prospective Register of Systematic Reviews (PROSPERO) (https://www.crd.york.ac.uk/PROSPERO/) with the registration number CRD42024535841.

## 1 Introduction

Osteoarthritis is a prevalent disabling disease with enormous impact on patients, the healthcare system and wider socio-economic costs, representing a significant and growing health burden (1, 2). Clinically, the knee is the most common location of osteoarthritis (3). Notably, knee osteoarthritis ranks as the 11th most prevalent condition out of the 291 diseases listed by the World Health Organization in terms of years lived with disability (4). Additionally, it imposes a substantial economic burden on society, contributing between 1.1 and 2.5% of the nation’s gross domestic product (5). In KOA, cartilage degeneration, joint space loss, bony encumbrances and subchondral bone changes all cause joint pain, stiffness, and reduced mobility (6). Although the precise cause of knee osteoarthritis remains incompletely understood, various factors including age, obesity, trauma, and genetics contribute to its development (7). Traditionally, radiography has been employed for diagnosing KOA (8). In terms of treatment, education, exercise, and weight loss constitute fundamental pillars of its management (9), complemented by oral medications, nutritional supplementation [e.g., chondroitin sulfate, glucosamine, vitamin D (10)], and physical therapy. However, in cases where patients experience severe symptoms and structural damage, surgery represents the optimal choice (9).

**Figure 1:**
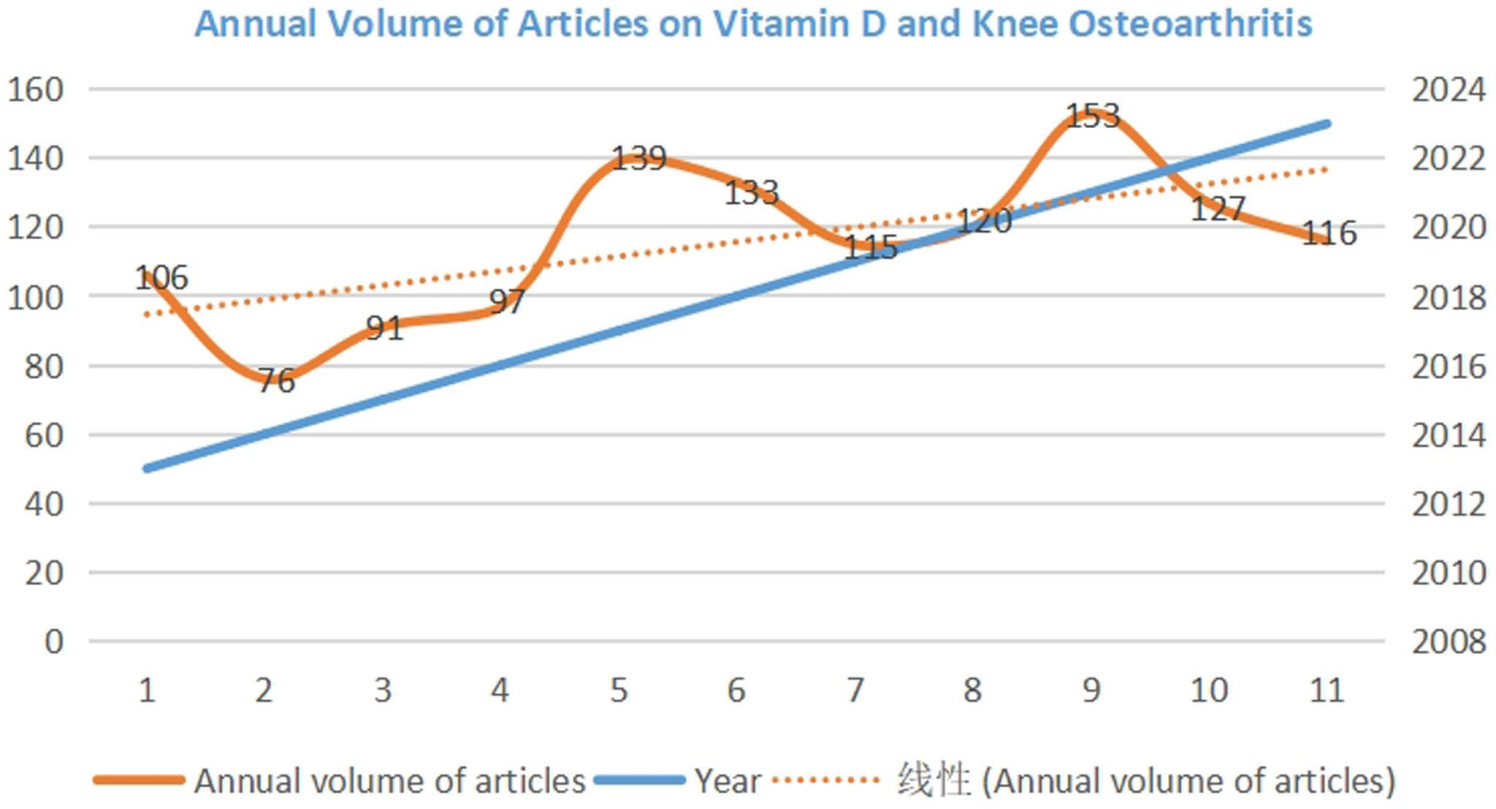
Annual volume of articles on vitamin D and knee osteoarthritis.

Over a century ago, the discovery that vitamin D supports bone growth was a major public health victory (11). In recent years, Vitamin D supplements cure osteoarthritis has garnered attention from medical professionals and patients due to its simplicity and portability. As in Figure 1, annual volume of articles on Vitamin D and Knee Osteoarthritis have risen annually over the past decade.

Health interventions are evaluated on the basis of systematic reviews and meta-analyses which rank highest in the ‘evidence hierarchy’ (11). Several current evidence-based studies demonstrate that vitamin D supplementation may be related to improved knee function and reduced pain in patients with osteoarthritis of the knee (12–14). While, several SRs/MAs have found no clear link between vitamin D supplementation and knee osteoarthritis (15–17). Relying solely on low-quality SR/MA with inconclusive conclusions is inadequate to guide clinical practice (12), so we conducted an overview of relevant SRs/MAs.

An overview is a collection of data from multiple systematic reviews that summarizes relevant evidence for decision-making (13). The purpose of the study was to search, gather, and analyze data on the characteristics and quantitative results of SR/MA in patients with KOA treated with Vitamin D supplementation, and objectively evaluate the efficacy of supplements. For future clinical studies, this overview provides clinical evidence and recommendations.

## 2 Method

This study provides an overview based on the PRISMA statement for conducting SR/MA (11). The program of this review is registered in the Prospective Registry of International Systematic Reviews (PROSPERO) under the number: CRD42024535841.

### 2.1 Inclusion criteria

This overview was based on the specified inclusion criteria:

#### 2.1.1 Studies

SRs/MAs of RCTs investigating vitamin D supplementation for KOA were included.

#### 2.1.2 Participants

A knee osteoarthritis diagnosis based on internationally accepted criteria does not take into account gender, age, geographical location, ethnicity, or disease duration.

#### 2.1.3 Interventions

The intervention group received vitamin D supplements as the primary intervention, without specific requirements regarding the type, dosage, or duration of vitamin D formulation.

The control group received placebo.

#### 2.1.4 Outcome measures

A WOMAC score, Visual Analog Scale (VAS), changes in tibial cartilage volume (TCV), Joint Space Width (JSW) and synovial fluid volume were the most important outcomes. Secondary outcomes included serum vitamin D levels, bone marrow lesions and et al.

### 2.2 Exclusion criteria

Following are the criteria for exclusion: (1) It is not a systematic review or meta-analysis specifically studying the use of vitamin D supplementation for KOA. (2) Studies primarily focusing on serum VD levels or VD-related genes. (3) Interventions targeted at patients with other forms of arthritis, excluding knee osteoarthritis. (4) Duplicate publications. (5) Network meta-analyses, conference abstracts, letters or narrative reviews.

### 2.3 Search strategy

In this study, two researchers (Zhang and Ye) conducted independent searches on four databases, namely PubMed, Embase, Web of Science and Cochrane Library, without restrictions on language or publication status. Literature searches were conducted using MeSH terms, keywords, and free-text terms like “vitamin D supplement,” “knee osteoarthritis” and “systematic review.” Additionally, screening of reference lists for articles in systematic reviews or meta-analyses, study registrations, and gray literature was performed to ensure comprehensive coverage. Comprehensive search strategies for all databases can be provided in Appendix 1.

### 2.4 Study selection

A third researcher resolved any discrepancies between the studies selected by two researchers working independently. All retrieved results were transferred into NoteExpress software to delete duplicates and irrelevant studies guided by the advanced protocol. Using the title and abstract of studies aligned with the study’s objectives, each researcher independently screened them. Subsequently, the complete texts of the chosen articles were reviewed according to the criteria set in advance to assess eligibility. For a list of excluded articles, please refer to Appendix 1.

### 2.5 Data extraction

For each included SR/MA, two independent researchers conducted data extraction. Selective data encompass first author, publication year, country, type of study, quantity of RCTs, population, sample size, search time and databases, quality assessment tools, experimental interventions, control interventions, outcome indicators and overall conclusions. The extracted data were cross-checked, and any discrepancies were resolved through discussion with a third researcher.

### 2.6 Quality assessment

Data extraction was followed by scoring each study based on the Cochrane risk of bias tool and Jadad score. Quality assessment focused on four areas: (1) methodological quality (2) risk of bias (3) reporting quality (4) evidence quality. Two researchers independently evaluated these four quality assessment methods. Before the assessment, in-depth discussions were conducted to reach a consensus on each evaluation tool’s relevant items. Disputes were resolved with the help of a third reviewer.

#### 2.6.1 Methodological quality assessment

The AMSTAR-2 methodological quality appraisal tool has been used to evaluate systematic reviews since 200,715. There are sixteen items, including seven critical ones and nine non-critical ones.

Independent evaluations were conducted by two reviewers, and cross-checks were conducted by the third reviewer for resolution of any discrepancies. Following the AMSTAR-2 evaluation criteria, there were three types of evaluations: “yes, ““partial yes, “and “no.” “Yes” was marked for content that fully matched an item. “Partial yes” was marked for content that partially matched. If there was no match, “no” was marked for content that did not match. No or one non-critical weakness in SR/MA was rated high quality, more than one non-critical weakness was rated moderate, one or more critical flaws was rated low, and more than one critical flaw was rated very low.

#### 2.6.2 Risk of bias assessment

ROBIS has been developed to grade reviews, which is easily used for assessing bias. The tool consists of three main stages: The first stage is an optional initial assessment of the relevance of the included studies to the review question of interest. The second stage consists of four domains with a total of 20 questions: identify and select studies, collect and appraise data, and synthesize and present the results. At the third stage, the risk of bias in interpretation of the results is assessed and any limitations uncovered at the second stage are considered. As a result, each risk level was ranked as “low risk,” “high risk,” and “unclear risk” based on the overall bias risk judgment (12). Two reviewers perform the assessment independently, and their evaluations are cross-validated. Any discrepancies are deliberated upon with a third reviewer to reach a resolution.

#### 2.6.3 Reporting quality assessment

PRISMA 2020 is a development of PRISMA 2009, providing updated reporting guidelines for SR/MA, incorporating advancements in the identification, selection, assessment, and integrated research methods. PRISMA 2020 consists of a total of 27 checklists, and the evaluation for each item was divided into: “yes,” “partial yes,” or “no.” Two reviewers conduct the evaluation independently, the evaluation is followed by a cross-check, and any disputes are discussed with the third reviewer before a decision is made (17).

#### 2.6.4 Quality of evidence

A GRADE guideline evaluates the strength of evidence by taking into account factors such as bias, inconsistency, indirectness, imprecision, and publication bias. Four levels of evidence quality are categorized as “high level,” “moderate level,” “low level,” and “very low level.” Evidence quality was independently assessed by two reviewers. Prior to conducting a formal assessment, the reviewers are trained to ensure that a consensus is reached prior to conducting the assessment. Two reviewers conducted independent assessments, resolving any differences through discussion. If needed, a third reviewer evaluated the assessment after these discussions (18).

#### 2.6.5 Overlap calculation of the reviews

The duplicated studies can potentially exaggerate the therapeutic effects and lead to similar conclusions in the meta-analysis (13). Hence, we utilized the Corrected Coverage Area (CCA) to evaluate the overlap of included studies (14).

The CCA percentage is calculated as N-r / r*c-r, where N is the total count of publications, r is the number of primary publications, and c is the number of reviews. CCA values of 0–5% represent “slight overlap,” 6–10% signify “moderate overlap,”11–15% denote “high overlap” and values above 15% indicate “very high overlap” (15). CCA index can be used to assess overlap between primary studies in different systematic reviews. The systematic reviews are arranged in ascending order based on their publication dates. As requested, the gray diagonal tiles represent the number of individual/total primary studies included in each review. For each outcome, apart from the joint national assessment at the outcome level, we have also created a citation matrix to address the issue of overlap.

## 3 Results

### 3.1 Literature selection

**Figure 2:**
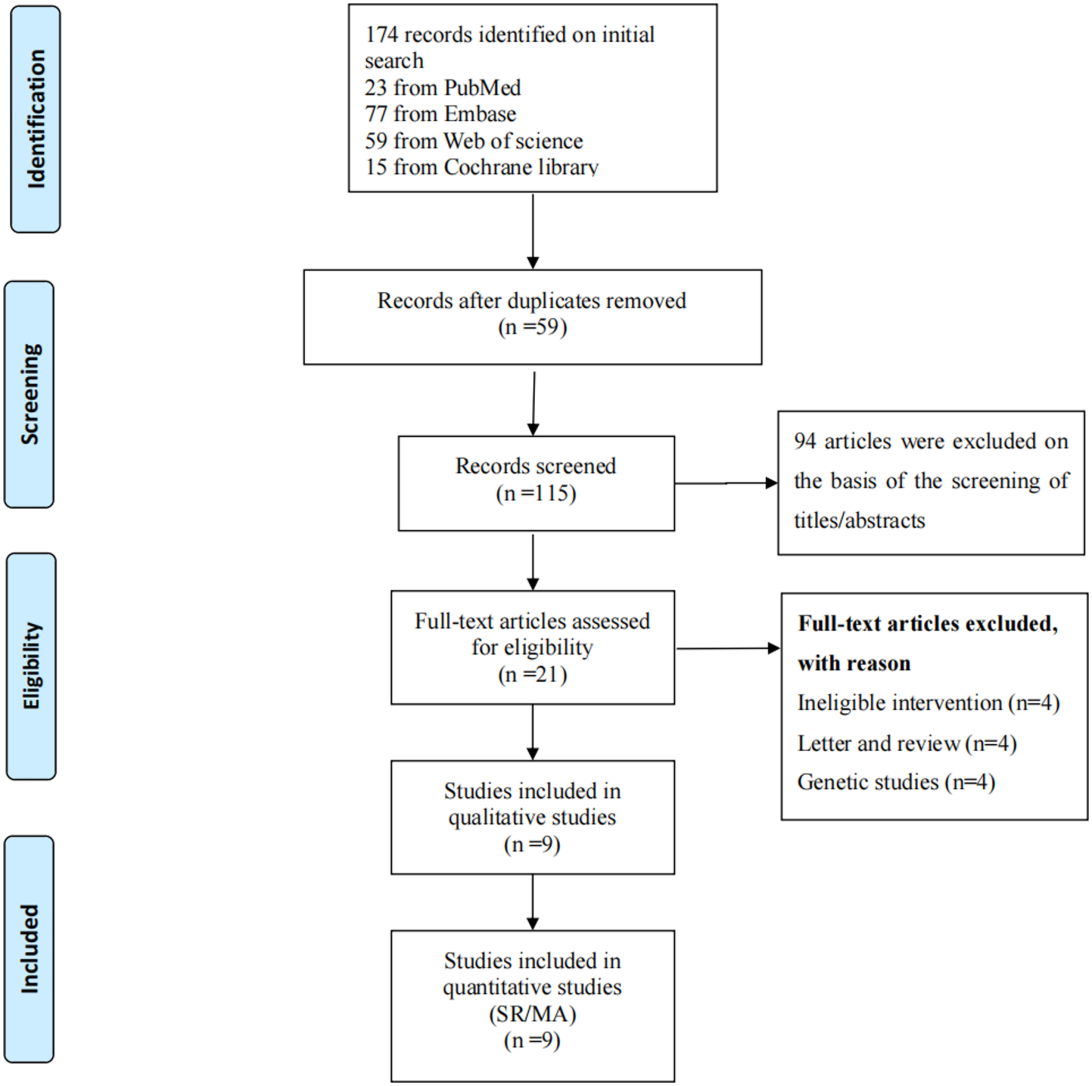
Search results (flow diagram).

174 articles were obtained by searching four databases, among which, 59 duplicates were deleted after importing into NoteExpress. Subsequently, 94 were removed based on title and abstract, and 21 relevant research papers were screening out. 12 papers were dropped after reading the full text based on our predetermined inclusion and exclusion criteria. Up to October 24th 2023, 9 papers were finally included. The detailed flow chart is shown in Figure 2 and the reasons for exclusion are given in Appendix.

### 3.2 Characteristics of the included SRs/mas

Databases searched included PubMed, Web of Science, Embase and Cochrane Library, and the time period was from the time of construction of the library until October 24, 2023. Eventually, we identified three SRs and six MA, whose publication years range from 2014–2023. These articles originated from China, France, the USA, and India, and were all published in English. The SRs/MA included RCTs with a combined sample size ranging from 146 to 3,077. The population was patients with KOA aged 45 years or older, there is no distinction between disease duration and severity. The Intervention group was treated with vitamin D supplements and the control group with placebo. The primary outcomes included the WOMAC, VAS, TCV, JSW etc. Secondary outcomes included serum vitamin D levels, bone marrow lesions, and synovial fluid volume. Seven studies selected appropriate tools to identify bias risks, which including the Cochrane bias assessment standards and the Jadad scale, while two papers did not report evaluation tools. Concert information is presented in Table 1.

**Table 1:** Characteristics of meta-analyses or systematic reviews evaluating the efficacy of vitamin D supplementation for KOA.

First author (year), country	Study type	Number of RCT studies	Populations	Sample size	Search time, included databases	Quality of included RCT	Experimental interventions	Control interventions	Outcome indicators	Overall conclusions
Wang (2023), China	MA	8	KOA patients	3,077	From inception to 23th Dec.2021 Pubmed, Embase, Cochrane	Cochrane criteria: a: 8 trials for low ROB b: 1/7 trials for high/low ROB c: 8 trials for low ROB d: 8 trials for low ROB e: 2/6 trials for high/low ROB f: 1/7 trials for high/low ROB g: 8 trials for low ROB	Vitamin D supplementation	Placebo	A; B; C; E; F; G; J; O	WOMAC pain, TCV, VAS, and synovial fluid volume are significantly reduced by vitamin D supplementation, but not JSW, bone marrow lesions, or WOMAC stiffness.
Mathieu (2022), France	MA	3	KOA patients	662	From inception to 18th Nov. 2021 Pubmed, Embase, Cochrane	Jadad scale criteria: 3 studies Jadad score = 5	Vitamin D supplementation	Placebo	B; C; D; E	Vitamin D supplementation is not find any significant improvement in total WOMAC or stiffness. However, improvement in VAS pain and WOMAC function is weak.
Zhao (2021), China	MA	6	KOA patients >45 years old	1,599	From inception to 15th Nov. 2020 PubMed, Embase, Web of science, Cochrane,CBM, Medline, CNKI, Wanfang, SinoMed	Cochrane criteria: a: 1/5 trials for unclear/high ROB b: 1/5 trials for unclear/high ROB c: 1/5 trials for unclear/high ROB d: 1/5 trials for unclear/high ROB e: 1/5 trials for unclear/high ROB f: 1/5 trials for unclear/high ROB g: 1/1/4 trials for low/unclear/high ROB	Vitamin D supplementation	Placebo	A; B; C; D; F; G; J; O	Vitamin D supplementation proves effective in alleviating WOMAC pain, WOMAC function, WOMAC stiffness, total WOMAC score, and synovial fluid volume. However, it did not yield a statistically significant impact on TCV, JSW, and bone marrow lesions.
Yu (2021), China	MA	4	KOA patients	1,130	From inception to Jul. 2019 Pubmed, Embase, Medline, Web of science, Google Scholar	None	Vitamin D supplementation	Placebo	A; F; G	Vitamin D supplementation is not significant effective in the change rates of the WOMAC pain scores, TCV and JSW.
Vaishya (2019), India	SR	2	KOA patients	249	From Jan.2005 to Dec.2015 Pubmed, Cochrane	None	Vitamin D supplementation	Placebo	A; B; E; K; L	There is limited evidence to support the use of vitamin D therapy in reducing cartilage volume loss and knee pain in KOA.
Hussain (2017), India	SR	5	KOA patients ≥45 years old	1,189	From inception to 6th Jul. 2016 PubMed,Embase, Cochrane CENTRAL	Cochrane criteria: a: 5 trials for low ROB b: 5 trials for low ROB c: 5 trials for low ROB d: 1/1/3 trials for unclear/high/low ROB e: 1/4 trials for unclear/low ROB f: 1/4 trials for high/low ROB g: 1/4 trials for unclear/low ROB	Vitamin D supplementation.	Placebo	A; B; C; D; E; F; H; J; M	Vitamin D supplementation has not been shown to reduce structural disease progression and improve KOA management in this systematic review.
Diao (2017), China	MA	4	KOA patients ≥50 years old	1,130	From inception to 22th Jan. 2017 Embase, Medline, Web of science	Cochrane criteria: a: 4 trials for low ROB b: 4 trials for low ROB c: 4 trials for low ROB d: 4 trials for low ROB e: 4 trials for low ROB f: 4 trials for low ROB g: 4 trials for low ROB	Vitamin D supplementation	Placebo	A; F; G	For patients with KOA, vitamin D supplementation had a statistical significant effect on WOMAC pain control, but to a lesser extent. However, TCV and JSW were unchanged.
Gao (2017), China	MA	4	KOA patients ≥60 years old	1,136	From inception to Dec.2016 Pubmed,Embase, Cochrane, Web of science	Cochrane criteria: a: 4 trials for low ROB b: 4 trials for low ROB c: 4 trials for low ROB d: 4 trials for low ROB e: 4 trials for low ROB f: 4 trials for low ROB g: 4 trials for low ROB	Vitamin D supplementation	Placebo	A; B; C; J; I; N	WOMAC pain and function can be improved by vitamin D supplements, but they do not reduce the progression of KOA.
Gallagher (2014), USA	SR	1	KOA patients	146	From inception to Jun. 2013 Pubmed, Embase, Cochrane	Jadad scale criteria: Jadad score = 5 Cochrane criteria: a: The trial for low ROB b: The trial for low ROB c: The trial for low ROB d: The trial for low ROB e: The trial for low ROB f: The trial for low ROB g: The trial for low ROB	Vitamin D, supplementation	Placebo	F; G	Vitamin D supplementation is no beneficial effect in JSW or TCV.

### 3.3 Methodology quality assessment

**Figure 3:**
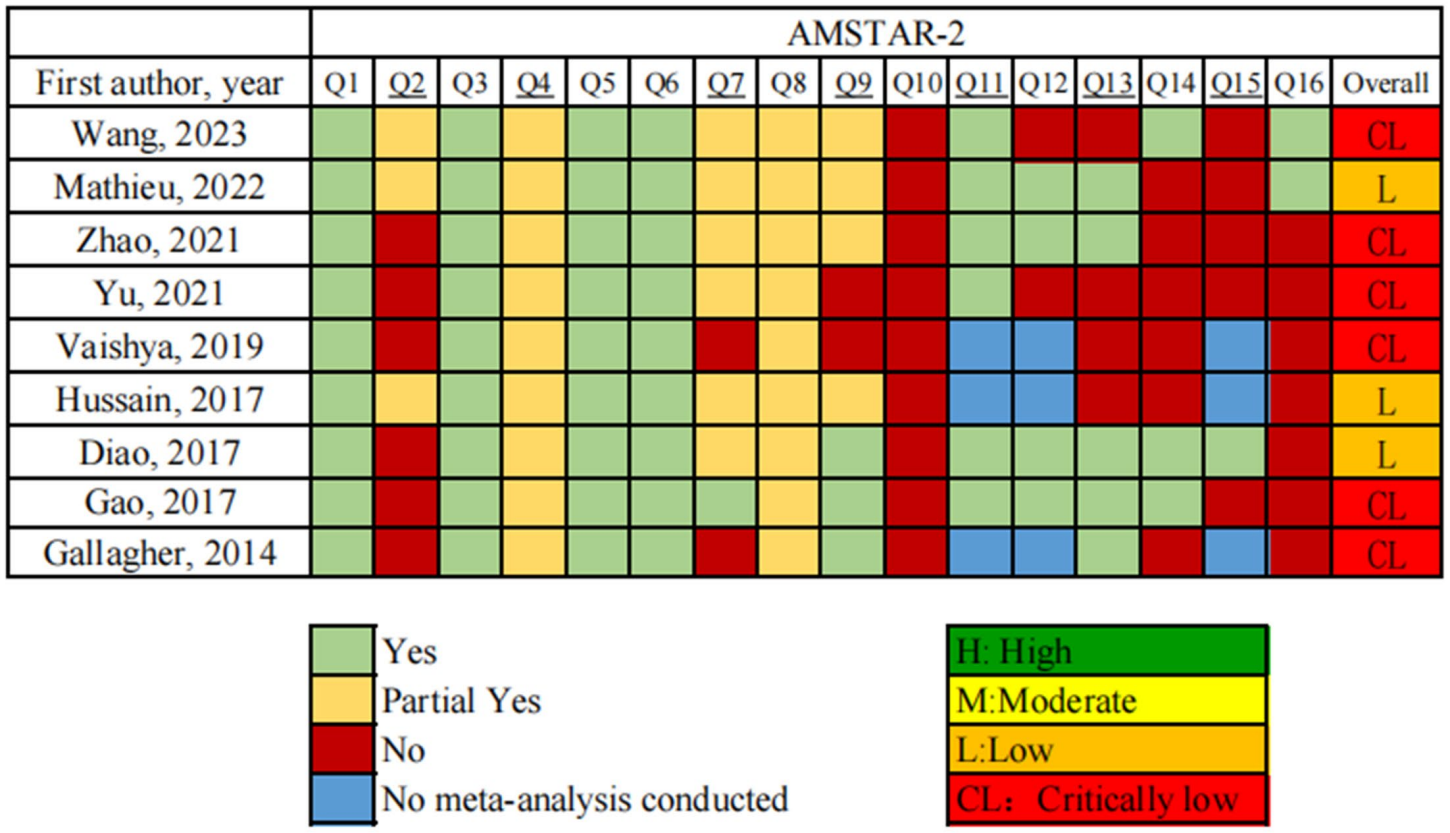
Results of the AMSTAR 2 assessments.

Our methodological quality assessment was conducted using AMSTAR 2.0. The quality assessment of three studies was deemed to be low, while the quality assessment of six studies was deemed to be very low. Throughout the literature, the PICOS principles on inclusion criteria and research questions were clearly described. And the type of study included was RCT, with only one study explaining the reasons for this. Three studies (16–18) completed registration and published protocols in advance. Four studies (17–20) developed a comprehensive literature search strategy, yet failed to investigate the gray literature. Only one (21) listed the reasons for literature exclusion, but have no list. Furthermore, RCT was delineated in tabular form based on its essential attributes. Besides, Zhao (22) reported the source of funding. Meta-analyses of RCTs have not always taken the risk of bias into consideration. While three articles (16, 18, 21) acknowledged the potential presence of publication bias, none of them delved into the implications of such bias on study outcomes. Three studies (20, 22, 23) employed funnel plots as a method to identify publication bias. However, since few studies were included in any of the above articles, statistical tests for publication bias were not conducted. Only two studies (16, 23) investigated the sources of heterogeneity. Creditably, there was no conflict of interest reported in any of the nine studies. A thorough evaluation report is shown in Figure 3.

### 3.4 Biased risk assessment

Based on ROBIS, the risk of bias of the included studies was assessed. In Stage 1 (assessment of relevance) and Domain 1 (selection of studies), nine studies were rated as low risk of bias. In Domain 2 (identification and selection of studies), four studies were rated as low risk of bias and five studies as high risk of bias. During the search process, the search databases were not comprehensive, and alternative search methods such as manual searching and reference tracing were not conducted to identify relevant studies. In Domain 3 (data extraction and quality assessment), four studies were rated as high risk of bias. Mathieu (18) used the Jaded quality assessment tool but did not include allocation concealment, which could overlook significant bias. One study (22) did not specify the allocation of quality assessors. In Domain 4 (data synthesis and presentation of results), three studies (16, 18, 21) were rated as high risk of bias due to factors including a small number of included studies, lack of reporting bias and sensitivity analysis, or failure to clearly discuss the impact of reporting bias on the results in the results or discussion sections of the article. Finally, Stage 3 involved judging the overall risk of bias in the systematic evaluation, with three articles (16, 18, 21) rated as high risk. Please see Table 2 for further details.

**Table 2:** Results of ROBIS assessment.

Study	Phase 1	Phase 2				Phase 3
	Relevance assessment	Domain 1. Criteria for study eligibility	Domain 2. Study identification and selection	Domain 3. Data collection and study appraisal	Domain 4. Conclusions and synthesis	Risk of bias in the review
Wang (2023)	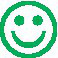	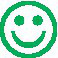	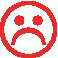	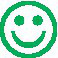	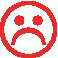	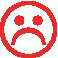
Mathieu (2022)	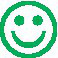	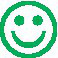	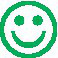	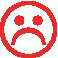	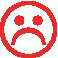	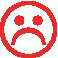
Zhao (2021)	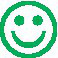	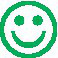	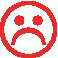	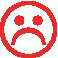	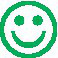	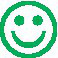
Yu (2021)	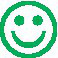	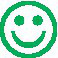	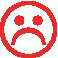	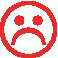	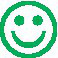	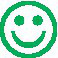
Vaishya (2019)	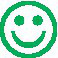	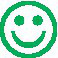	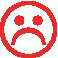	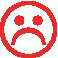	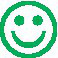	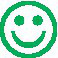
Hussain (2017)	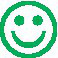	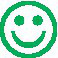	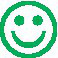	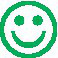	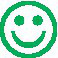	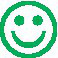
Diao (2017)	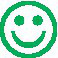	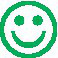	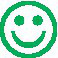	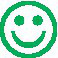	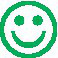	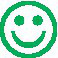
Gao (2017)	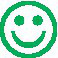	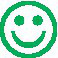	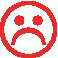	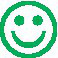	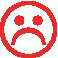	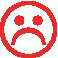
Gallagher (2014)	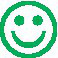	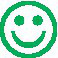	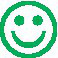	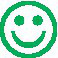	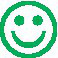	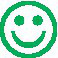

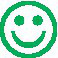
￼: low risk ￼: high risk.

### 3.5 Reporting quality assessment

**Figure 4:**
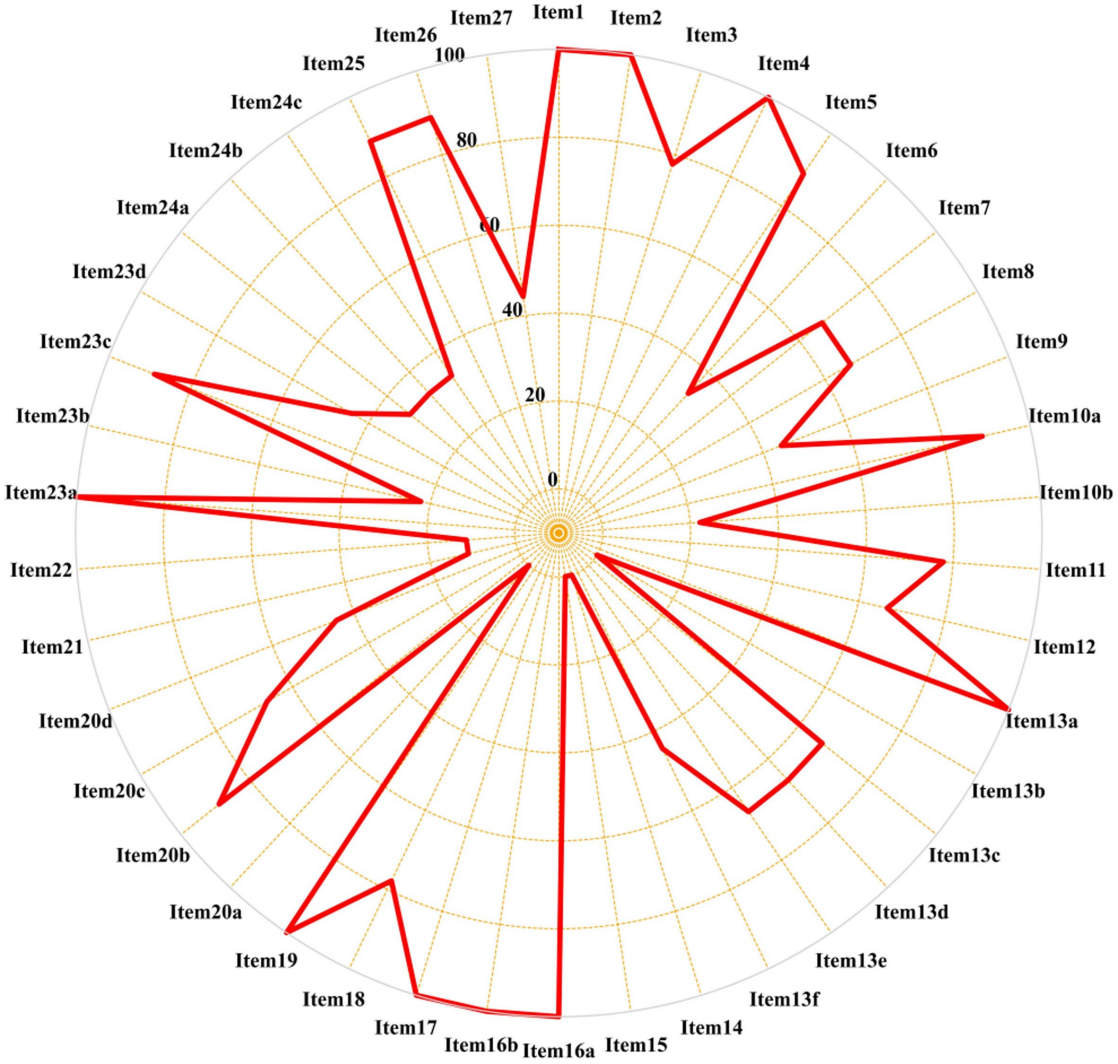
Results of the PRISMA 2020 assessments.

The PRISMA 2020 report involves 7 sections, encompassing 27 items (42 sub-items). An assessment of the reporting quality for PRISMA 2020 can be seen in Figure 4. Notably, 19 sub-items displayed compliance below the 60% threshold. Some studies were deficient in providing comprehensive information on database sources (Q6: 44.4%), while others neglected to report personnel allocation during data collection (Q9: 55.6%). Furthermore, certain studies omitted to elucidate any assumptions regarding missing or ambiguous data in their extractions (Q10b: 22.2%). Surprisingly, none of the reviews acknowledged the pre-processing of merged data (Q13b: 0%). Additionally, a proportion of studies failed to conduct sensitivity analysis of their assessed results (Q13f: 33.3%), nor did they address reporting bias (Q14: 33.3%). Alarmingly, only one study assessed evidence quality (Q15: 11.1%). Curiously, none of the reviews reported the bias risk associated with each synthesized result (Q20a: 0%). Two studies discussed potential sources of heterogeneity in the results (Q20c: 22.2%). Furthermore, some studies neglected to assess the robustness of their synthesized results through sensitivity analysis (Q20d: 33.3%). Surprisingly, none of the studies evaluated the bias risk due to missing outcomes in each synthesized result (Q21: 0%). Astonishingly, only one study presented the assessment results of evidence quality grading for each outcome indicator. Remarkably, certain studies overlooked discussing the limitations stemming from evidence bias in their discussions (Q23b: 22.2%). Moreover, some studies failed to address the impact of their results on future research (Q23d: 44.4%). Encouragingly, only three studies (16–18) completed pre-registration and published their protocols before conducting the studies (Q24a: 44.4; Q24b: 44.4; Q24c: 44.4%). Additionally, four studies made their data, code, or other materials publicly available (Q27: 44.4%), as outlined in Appendix 2.

### 3.6 Evidence quality assessment

Using a GRADE system, we assessed the quality of evidence related to SR/MA outcomes. Table 3 illustrates the evidence quality for 29 outcome indicators. A moderate quality of evidence was found for five outcomes, a low quality of evidence for 14 outcomes and a very low quality of evidence for 10 outcomes. As a result of publication bias and imprecision, evidence quality was lowered the most, followed by inconsistency and publication bias. Following is a breakdown of reasons for lowering evidence quality: risk of bias (25/45, 55.6%), inconsistency (14/45, 31.1%), imprecision (17/45, 37.8%), and publication bias (8/45, 17.8%).

**Table 3:** Results of GRADE on evidence of included meta-analyses or systematic reviews.

Outcome	Study	Intervention	Included RCTs (A/B)	Effect (95%CI)	*I* ^2^	*p* value	Quality assessment					Quality of evidence
							Risk of bias	Inconsistency	Indirectness	Imprecision	Publication bias	
Total WOMAC	Mathieu (2022)	Vitamin D 50,000–60,000 IU/m vs. Placebo	2RCT (516/261)	SMD −0.92 (−2.32, 0.48)	97%	0.00	0	−1^b^	0	−1^c^	−1^d^	Very low
	Zhao (2021)	Vitamin D 800–6,000 IU/d vs. Placebo	3RCT (497/487)	SMD −0.67 (−1.23, −0.12)	94%	0.02	0	−1^b^	0	−1^c^	−1^d^	Very low
WOMAC pain	Wang (2023)	Vitamin D 800–60,000 IU/d vs. Placebo	6RCT (1,305/1309)	SMD −0.11 (−0.18, −0.03)	94%	0.007	−1^a^	−1^b^	0	0	0	Low
	Zhao (2021)	Vitamin D 800–6,000 IU/d vs. Placebo	4RCT (570/560)	SMD −0.32 (−0.63, −0.02)	82%	0.04	0	−1^b^	0	0	−1^d^	Low
	Yu (2021)	Vitamin D 800–60,000 IU/d vs. Placebo	4RCT (570/560)	SMD −1.18 (−2.81, 0.44)	99.2%	0.00	NR	−1^b^	0	0	NR	Moderate
	Diao (2017)	Vitamin D 800–60,000 IU/d vs. Placebo	4RCT (570/560)	SMD −0.32 (−0.63, −0.02)	82%	0.0007	0	−1^b^	0	0	−1^d^	Low
	Gao (2017)	Vitamin D 800–60,000 IU/d vs. Placebo	4RCT (571/565)	WMD −1.65 (−2.16, −1.14)	0%	0.473	0	0	0	0	−1^d^	Moderate
WOMAC stiffness	Wang (2023)	Vitamin D ≥ 50,000/m vs. Placebo	4RCT (995/999)	SMD −0.52 (−1.07, 0.03)	95%	0.06	−1^a^	−1^b^	0	0	−1^d^	Very low
	Mathieu (2022)	Vitamin D 50,000–60,000 IU/m vs. Placebo	2RCT (516/261)	SMD −0.07 (−0.25, 0.10)	0%	0.47	0	0	0	0	−1^d^	Moderate
	Zhao (2021)	Vitamin D 800–6,000 IU/d vs. Placebo	3RCT (497/487)	SMD −0.13 (−0.26, −0.01)	21%	0.04	0	0	0	−1^c^	−1^d^	Low
	Gao (2017)	Vitamin D 800–60,000 IU/d vs. Placebo	3RCT (498/492)	WMD 0.03 (−0.17, 0.24)	53.4%	0.117	0	0	0	−1^c^	−1^d^	Low
WOMAC function	Wang (2023)	Vitamin D ≥ 50,000/m vs. Placebo	5RCT (1,068/1072)	SMD −0.88 (−1.47, −0.29)	96%	0.004	−1^a^	−1^b^	0	0	0	Low
	Mathieu (2022)	Vitamin D 50,000–60,000 IU/m vs. Placebo	3RCT (662/334)	SMD −0.44 (−0.80, −0.09)	76%	0.015	0	−1^b^	0	0	−1^d^	Low
	Zhao (2021)	Vitamin D 800–6,000 IU/d vs. Placebo	4RCT (570/560)	SMD −0.34 (−0.60, −0.08)	75%	0.01	0	−1^b^	0	0	−1^d^	Low
	Gao (2017)	Vitamin D 800–60,000 IU/d vs. Placebo	4RCT (571/565)	WMD −1.87 (−2.58, −1.17)	47.6%	0.126	0	0	0	0	−1^d^	Moderate
Visual analog scale	Wang (2023)	Vitamin D 50,000–640,000 IU/m vs. Placebo	3RCT (321/275)	SMD −0.32 (−0.48, −0.15)	45%	0.0002	−1^a^	0	0	−1^c^	−1^d^	Very low
	Mathieu (2022)	Vitamin D 50,000–60,000 IU/m vs. Placebo	3RCT (662/334)	SMD −0.20 (−0.35, −0.04)	0%	0.926	0	0	0	0	−1^d^	Moderate
Tibia cartilage Volume	Wang (2023)	Vitamin D ≥ 50,000 IU/m vs. Placebo	2RCT (282/277)	SMD 0.18 (0.01, 0.34)	0%	0.04	−1^a^	0	0	−1^c^	−1^d^	Very low
	Zhao (2021)	Vitamin D 50,000–60,000 IU/m vs. Placebo	2RCT (282/277)	SMD 0.12 (−0.05, 0.29)	0%	0.15	0	0	0	−1^c^	−1^d^	Low
	Yu (2021)	Vitamin D ≥ 50,000 IU/m vs. Placebo	2RCT (282/277)	SMD 0.10 (−0.07, 0.27)	34.1%	0.218	NR	0	0	−1^c^	−1^d^	Low
	Diao (2017)	Vitamin D ≥ 50,000 IU/m vs. Placebo	2RCT (282/277)	SMD 0.12 (−0.05, 0.29)	0%	0.66	0	0	0	−1^c^	−1^d^	Low
Joint space width	Wang (2023)	Vitamin D 800–8000 IU/d vs. Placebo	2RCT (310/310)	SMD 0.02 (−0.24, 0.28)	52%	0.88	−1^a^	−1^b^	0	−1^c^	−1^d^	Very low
	Zhao (2021)	Vitamin D 800–2000 IU/d vs. Placebo	3RCT (306/303)	SMD −0.10 (−0.26, 0.05)	2%	0.2	0	0	0	−1^c^	−1^d^	Low
	Yu (2021)	Vitamin D 800–8000 IU/d vs. Placebo	2RCT (310/310)	SMD 1.58 (−1.82, 4.98)	99.6%	0.00	NR	−1^b^	0	−1^c^	NR	Low
	Diao (2017)	Vitamin D 800–8000 IU/d vs. Placebo	2RCT (309/305)	SMD 0.07 (−0.08, 0.23)	59%	0.12	0	−1^b^	0	−1^c^	−1^d^	Very low
Bone marrow lesions	Wang (2023)	Vitamin D 800–8000 IU/d vs. Placebo	3RCT (306/303)	SMD −0.16 (−0.31, 0.00)	38%	0.06	−1^a^	0	0	−1^c^	−1^d^	Very low
	Zhao (2021)	Vitamin D 800–2000 IU/d vs. Placebo	2RCT (309/305)	SMD 0.03 (−0.26, 0.31)	59%	0.85	0	−1^b^	0	−1^c^	−1^d^	Very low
Synovial fluid volume	Wang (2023)	Vitamin D 2400–50,000 IU/m vs. Placebo	2RCT (233/230)	SMD 0.20 (0.02, 0.38)	0%	0.03	−1^a^	0	0	−1^c^	−1^d^	Very low
	Zhao (2021)	Vitamin D 2400–50,000 IU/m vs. Placebo	2RCT (233/230)	SMD −0.20 (−0.39, −0.02)	0%	0.03	0	0	0	−1^c^	−1^d^	Low

### 3.7 Overlap of the included SRs/MAs

**Figure 5:**
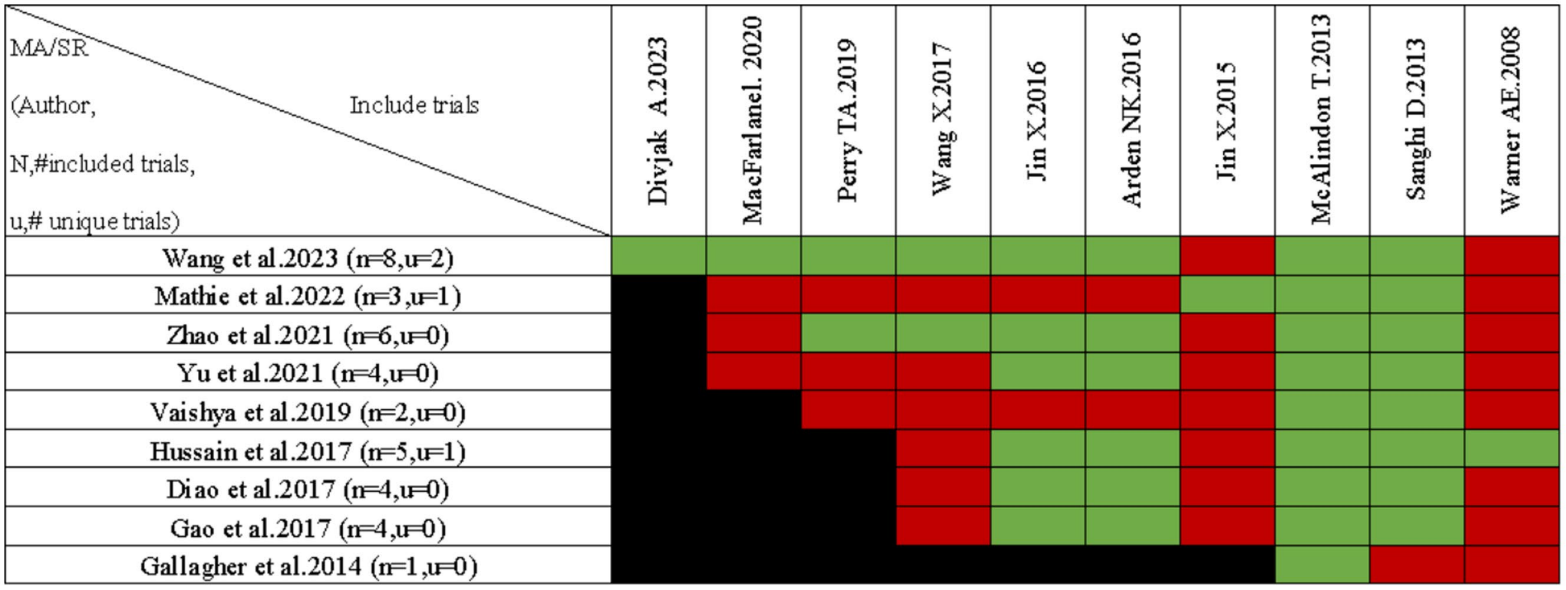
Citation matrix for reviews reporting vitamin D supplementation for knee osteoarthritis.

**Figure 6:**
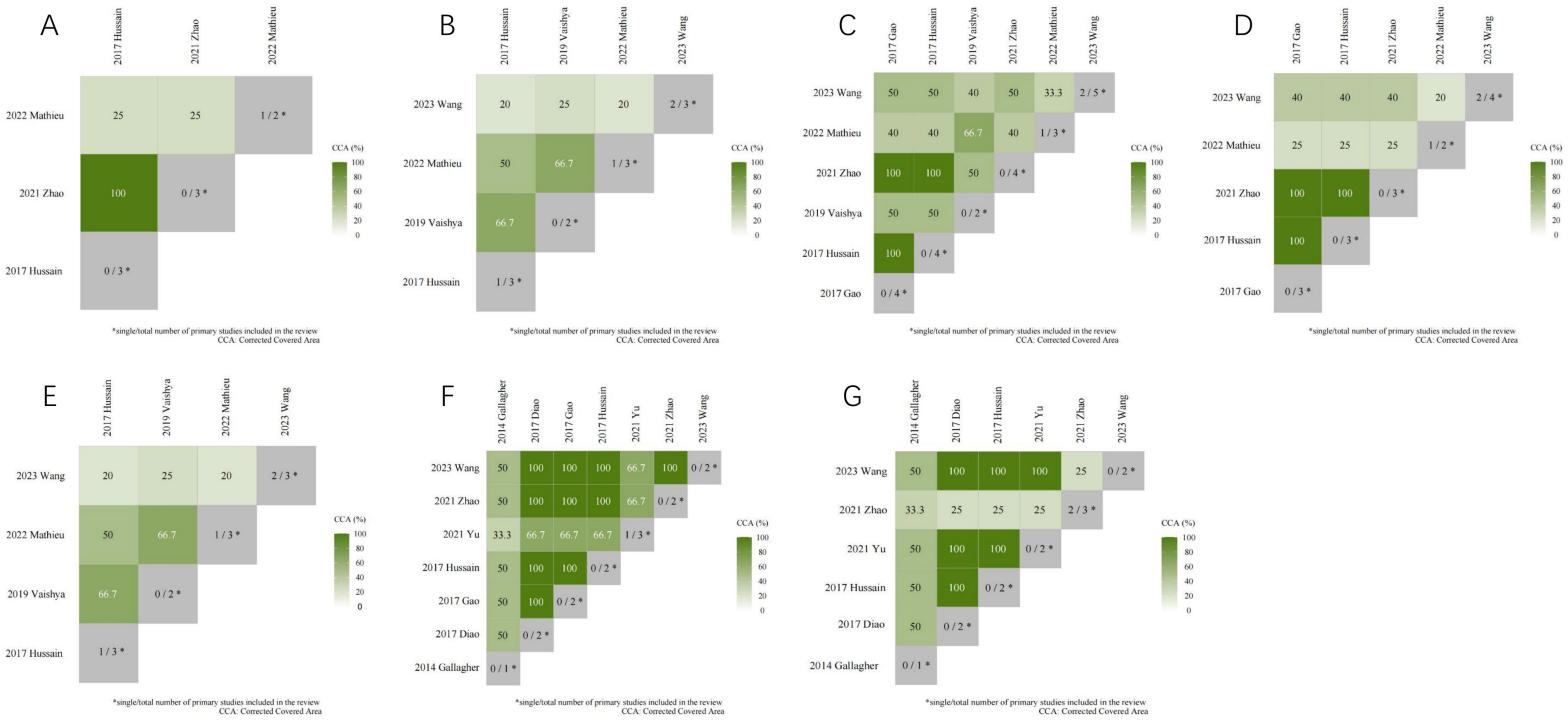
The heatmap of the overlap between the studies included in the systematic review of vitamin D supplements in treating KOA.

A citation matrix for vitamin D supplementation for the treatment of KOA was presented. Detailed evaluation results are shown in Figure 5. Data were derived from 10 included studies, with significant overlap in the primary studies included in 9 of them. The heatmap in Figure 6 illustrates the overlap between the studies included in the systematic review of vitamin D supplements in treating KOA. Listed in ascending order by date of publication are the systematic reviews. According to the figure, the gray diagonal tiles represent quantity of individual/total primary studies included in every review. For each outcome, apart from the joint national assessment at the outcome level, we have also created a citation matrix to address the issue of overlap. We will present the results through visualization, detailed in the Appendix. Figure 6A displays a heatmap representation of studies that overlap in systematic reviews for the Total WOMAC result, showing CCA values from 25 to 100%. Figure 6B presents a heatmap visualization of overlapping studies included in systematic reviews for the WOMAC pain outcome, with CCA values ranging from 20 to 66.7%. Figure 6C showcases a heatmap visualization of overlapping studies included in systematic reviews for the WOMAC function outcome, with CCA values ranging from 33.3 to 100%. Figure 6D displays a heatmap visualization of overlapping studies included in systematic reviews for the WOMAC stiffness outcome, with CCA values ranging from 20 to 100%. Figure 6E exhibits a heatmap visualization of overlapping studies included in systematic reviews for the VAS outcome, with CCA values changing from 20 to 66.7%. Figure 6F demonstrates a heatmap visualization of overlapping studies included in systematic reviews for the TCV outcome, with CCA values ranging from 33.3 to 100%. Figure 6G portrays a heatmap visualization of overlapping studies included in systematic reviews for the JSW outcome, with CCA values ranging from 25 to 100%. In general, several outcomes show substantial overlap, for further details, please see Appendix.

## 4 Effects of vitamin D supplementation interventions

### 4.1 Total WOMAC score

Total WOMAC scores were compared between vitamin D supplementation and placebo in two articles (18, 22). The results indicate that vitamin D supplements significantly improved WOMAC scores. (SMD = −0.92, 95% CI [−2.32, 0.48], *p* = 0.00; SMD = −0.67, 95% CI [−1.23, −0.12], *p* = 0.02).

### 4.2 WOMAC pain score

In five articles (16, 20–23), vitamin D supplementation was compared to placebo on WOMAC pain scores. The results indicate that four reviews (16, 20, 22, 23) demonstrated a significant impact of vitamin D supplementation on WOMAC pain scores (SMD = −0.11, 95% CI [−0.18, −0.03], *p* = 0.007; SMD = −0.32, 95% CI [−0.18, −0.03], *p* = 0.04; SMD = −1.18, 95% CI [−2.81, 0.44], *p* = 0.00; SMD = −0.32, 95% CI [−0.63, −0.02], *p* = 0.0007), while according to one review (21), vitamin D supplementation did not significantly improve WOMAC pain scores (WMD = −1.65, 95% CI [−2.16, −1.14], *p* = 0.473).

### 4.3 WOMAC stiffness score

In four articles (16, 18, 21, 22), VD supplements and placebo were compared in terms of stiffness scores using the WOMAC scale. The results indicate that only one review (22) revealed a notable effect of vitamin D supplementation on WOMAC stiffness scores (SMD = −0.13, 95% CI [−0.26, −0.01], *p* = 0.4). The remaining three reviews (16, 18, 21) found no substantial difference in WOMAC stiffness scores between vitamin D supplementation and placebo treatments (SMD = −0.52, 95% CI [−1.07, 0.03], *p* = 0.06; SMD = −0.07, 95% CI [−0.25, 0.10], *p* = 0.47; WMD = 0.03, 95% CI [−0.17, 0.24], *p* = 0.117).

### 4.4 WOMAC function score

In four articles (16, 18, 21, 22), VD supplements and placebo were compared in terms of function scores using the WOMAC scale. The findings reveal that three of the reviews (16, 18, 22) demonstrated a notable improvement in WOMAC function scores with vitamin D supplementation (SMD = −0.88, 95% CI [−1.47, −0.29], *p* = 0.004; SMD = −0.44, 95% CI [−0.80, −0.09], *p* = 0.015; SMD = −0.34, 95% CI [−0.60, −0.08], *p* = 0.01). Conversely, one review (21) reported no significant improvement (WMD = −1.87, 95% CI [−2.58, −1.17], *p* = 0.126).

### 4.5 Visual Analog Scale

In two articles (16, 18), vitamin D supplementation and placebo were compared in VAS. The findings indicate that Wang’s review reported a significant improvement with vitamin D supplementation (SMD = −0.32, 95% CI [−0.48, −0.15], *p* = 0.0002), whereas Mathieu’s review found no significant difference between vitamin D supplementation and placebo treatments (WMD = −0.20, 95% CI [−0.35, −0.04], *p* = 0.926).

### 4.6 Tibia cartilage volume score

In four articles (16, 20, 22, 23), VD supplements and placebo were compared in Tibia cartilage Volume scores. The results indicate that only one review demonstrated a significant improvement with vitamin D supplementation (SMD = 0.18, 95% CI [0.01, 0.34], *p* = 0.04). However, three reviews (20, 22, 23) found no significant difference in Tibia cartilage Volume scores between vitamin D supplementation and placebo treatments (SMD = 0.12, 95% CI [−0.05, 0.29], *p* = 0.15; SMD = 0.10, 95% CI [−0.07, 0.27], *I^2^* = 34.1%, *p* = 0.218; SMD = 0.12, 95% CI [−0.05, 0.29], *p* = 0.66).

### 4.7 Joint space width score

In four articles (16, 20, 22, 23), vitamin D supplementation and placebo were compared in Joint space width scores. The findings indicate that one review (23) reported a significant impact of vitamin D supplementation on Joint space width scores (SMD = 1.58, 95% CI [−1.82, 4.98], *p* = 0.00). However, three other reviews (16, 20, 22) did not find any significant difference in Joint space width scores between vitamin D supplementation and placebo treatments (SMD = 0.02, 95% CI [−0.24, 0.28], *p* = 0.88; SMD = −0.10, 95% CI [−0.26, 0.05], *p* = 0.2; SMD = 0.07, 95% CI [−0.08, 0.23], *p* = 0.12).

### 4.8 Bone marrow lesions score

In two articles (16, 22), vitamin D supplementation and placebo were compared in Bone Marrow Lesions. Unfortunately, both reviews concluded that there was no significant difference in BML between the vitamin D supplementation and placebo groups (SMD = −0.16, 95% CI [−0.31, 0.00], *p* = 0.06; SMD = 0.03, 95% CI [−0.26, 0.31], *p* = 0.85).

### 4.9 Synovial fluid volume score

In two articles (16, 22), vitamin D supplementation and placebo were compared in Synovial Fluid Volume Score. Fortunately, both reviews found a significant improvement (SMD = 0.20, 95% CI [0.02, 0.38], *p* = 0.03; SMD = −0.20, 95% CI [−0.39, −0.02], *p* = 0.03).

### 4.10 Adverse events

In the analysis of 9 studies, adverse events were reported in only two (17, 22). According to the first study (17), the incidence rates of adverse events and severe adverse events in the vitamin D supplementation group were 31.2 and 14.9%, respectively, while in the placebo group, they were 28.6 and 10.8%. Gao’s report (21) indicated that 27% of patients in the treatment group and 18% in the placebo group experienced at least one adverse event. There was no significant discrepancy between the placebo and vitamin D supplementation groups in the incidence rates of adverse events, according to these comprehensive analyses. Common adverse events in both groups included hypercalcemia, hypercalciuria and fractures.

## 5 Discussion

### 5.1 Main finding summary

This overview analyzed three SRs and six MA published between 2014 and 2023, including 10 RCTs with a total of more than 10,000 patients. To evaluate the methodological quality, risk of bias, quality of reporting and quality of evidence, we used the AMSTAR-2, PRISMA 2020, ROBIS and GRADE tools.

It was revealed by AMSTAR-2 that the methodological quality of the SRs included in this study is not promising, with three articles of low quality (33.3%, 3/9) and six studies of critically low quality. PRISMA 2020 found relatively optimistic reporting quality among the SRs studied in this study. In the assessment of evidence quality using GRADE, only 17.2% of outcome indicators were deemed to have moderate quality. Rating methodological quality presented the following problems: (1) Although all studies followed the PICOS principles, some did not specify the research protocol and provide the registration information before the SRs began. Protocols and reports can reduces deviation, enhances quality and conserves research resources as well (24). Other SRs authors can search the registration platform to determine if the study is duplicated (25). (2) All reviews included only RCT, but commonly did not explain the rationale for using this study design (26). (3) Very few studies have been able to satisfy a comprehensive literature search strategy. Most of the studies lacked a complete search strategy, i.e., consulting the reference list of the study, searching for pertinent grey literature, etc. Grey literature reduces publication bias, improves the completeness and currency of the review, and promotes a balanced understanding of the existing evidence (27). (4) According to the findings of ROBIS, Domain 2–4 of Phase-II had considerable problems, which comprises in selecting and searching studies, examining synthesis and findings. In particular, problems that arose in the Phase II study were not adequately explained, leading to a high risk of bias in the Phase III study. There is largely stemming from methodological limitations within the included original studies. These limitations encompass various biases in study design, implementation, and measurement, with insufficient detailing of randomization, allocation concealment, and blinding techniques (28). (5) There is a lack of complete lists of excluded studies in the majority of studies, which may have led to omissions during literature screening. Comprehensive retrieval of all relevant evidence and list reasons for elimination are an inherent component and challenge of a systematic review. (6) Risk of bias is used to check the potential reliability of the resulting evidence in a study (29). A prevalent issue is the high rate of publication bias and heterogeneity. Due to the limited amount of RCTs, coupled with the absence of statistical tests for publication bias or asymmetry in funnel plots, there exists the potential for bias in outcome indicators (30). Publication bias is instrumental in determining the comprehensiveness of relevant literature meeting systematic review inclusion criteria, including the retrieval of grey literature. Common testing methods for publication bias include funnel plots and Begg’s test and Egger’s test (31). (7) Truly, time and funding are barriers to conducting systematic reviews (32). Since no funding sources were reported in the reviews, it is not possible to assess the final results for objectivity.

The presentation of citation matrix and heat map revealed a significant overlap and consensus in research on the use of Vitamin D supplementation for treating KOA, although notable discrepancies were observed in certain outcome indicators. Exactly, there is some overlap between multiple SRs and this may lead to an overestimation of quality.

The above results indicate that there is a potential advantages of Vitamin D supplementation in improving Total WOMAC scores and synovial fluid volume in KOA. However, for this conclusion to be justified, more high-quality RCTs or MA/SR are needed.

### 5.2 Selection of KOA outcomes

KOA is a chronic disease characterized by persistent knee joint pain and functional decline (33). It involves not only mechanical degradation of joint cartilage but also structural and functional changes in the entire joint, including the synovium, meniscus, periarticular ligaments, and subchondral bone. It is an inflammatory disease affecting the entire synovial joint (34). Therefore, in selecting outcomes, we primarily focused on three major categories: pain-related assessment scales, knee joint function measures, and adverse event rates. As pain and knee joint dysfunction are the core symptoms of KOA patients, pain-related assessment scales are the preferred method for evaluating treatment efficacy. Among these, VAS has multiple uses in health and healthcare, such as for measuring pain and providing a single index measure of health-related quality of life (HRQoL) (35). It has been widely used in KOA studies because of their convenience, simplicity, ease of understanding, and suitability for telephone follow-up. The WOMAC scale, which assesses pain, stiffness, and physical function, is also commonly used and effectively evaluates disease progression and treatment effects in KOA patients (36, 37). Additionally, we paid particular attention to parameters such as Tibia Cartilage Volume score (38), Joint Space Width score (39), Bone Marrow Lesions score (40), and Synovial Fluid Volume score (41), all of which reflect changes in knee joint structure to a certain extent, indicating the alleviation or worsening of knee osteoarthritis. With the advancement of society, people have increasingly higher expectations for their health status and quality of life. They not only seek symptomatic relief from medications but also consider the potential impact of side effects on their daily lives. As a result, we also investigated the occurrence rate of adverse events associated with vitamin D supplementation.

### 5.3 The mechanism of KOA with vitamin D supplements and other combination therapies

Vitamin D affects joint health through a number of mechanisms, including maintaining calcium homeostasis, enhancing bone metabolism by promoting calcium absorption, and regulating chondrocyte function (42). It exists in two primary forms: D2 (ergocalciferol) and D3 (cholecalciferol), with D3 being synthesized in the skin upon UVB exposure. Cholecalciferol is biologically inactive and must undergo hydroxylation in the liver and kidneys to form 1α,25-dihydroxyvitamin D3 (1α,25(OH)_2_D3) (43). Vitamin D acts through the vitamin D receptor (VDR) to regulate circulating calcium and phosphate homeostasis by altering renal reabsorption and intestinal absorption. In knee osteoarthritis (KOA), VDR exerts an anti-inflammatory effect by modulating bone and cartilage metabolism, local inflammation, and vitamin D regulation of cartilage integrity and calcium content, and by inhibiting pro-inflammatory cytokines, such as TNF-alpha and IL-6, which affects an individual’s susceptibility to KOA (42). Given these biological mechanisms by which vitamin D influences joint health, its potential therapeutic role in knee osteoarthritis (KOA) has garnered increasing attention in recent years, particularly in relation to its ability to alleviate symptoms and improve joint function.

The management of knee osteoarthritis requires a multifaceted approach, integrating vitamin D supplementation, exercise, pain relief medications, and muscle relaxants. Various forms of exercise (including high-intensity resistance training) —such as aerobic, strength, balance, and aquatic training—are effective in reducing inflammation, slowing cartilage degeneration, and improving tendon and muscle function (44, 45). Home-based circuit training has also shown substantial benefits, improving musculoskeletal health and quality of life (46). Pain management is essential in treating KOA, with non-steroidal anti-inflammatory drugs (NSAIDs) or analgesics commonly prescribed to alleviate pain and reduce inflammation. These analgesic drugs reversibly bind to hydrophilic vesicles near the COX-2 activation site and can inhibit the conversion of arachidonic acid to prostaglandin H2, resulting in anti-inflammatory and analgesic effects (47). Muscle relaxants, frequently used to address muscle spasms and stiffness associated with OA, help reduce pain and improve mobility, enabling better engagement in exercise (48). By targeting different aspects of the disease, the combination of exercise, pain medications, and muscle relaxants offers a comprehensive approach to managing KOA. These interventions, along with vitamin D supplementation, achieve a therapeutic effect where “1 + 1>2,” promote bone health, reduce inflammation, and support joint repair.

### 5.4 Implications for future studies

Taking this overview into account, we have formulated the following recommendations for future research. Firstly, whether it is an SR/MA or an RCT, program and registration must be drafted to reduce potential bias and minimize the need for extensive revisions. Secondly, a comprehensive and elaborate search strategy along with indexing criteria should be formulated. The inclusion of references in the study, as well as the retrieval of grey literature, is particularly important. The process of study selection should provide a list of excluded literature to facilitate the monitoring of omissions. Thirdly, it is crucial to clarify the reasoning for included RCTs and describe the characteristics in detail, while also strictly adhering to the principles of multiple independent assessments, duplication, rigorous screening, and data extraction. Based on this foundation, the quality of SR/MA can be improved. Fourthly, utilizing suitable statistical methodologies to amalgamate findings and accounting for the influence of bias risk from individual RCTs on the overall outcome is imperative. In addition, in the presence of significant heterogeneity, conducting subgroup analysis or meta-regression analysis is essential to further elucidate the sources of heterogeneity. In CCA, overlapping research reflects unnecessary duplication. It is recommended to use funnel plots and perform Begg’s and Egger’s tests to detect publication bias whenever possible. All studies must clearly disclose their sources of funding and any potential conflicts of interest.

Analysis of adverse reactions has shown that excessive vitamin D supplementation can lead to hypercalcemia and an increased risk of fractures. Therefore, we recommend regular review of blood calcium, bone metabolism markers, and bone density when using Vitamin D supplements to adjust the dosage or intermittent administration according to the patient’s actual condition and personalize the treatment plan (49). In a short, we emphasized the importance of well-designed clinical trials to determine the optimal dosage range, taking into account individual factors such as baseline vitamin D levels, and risk of adverse effects like hypercalcemia and fractures. This forward-looking perspective aims to guide future research toward providing actionable and evidence-based recommendations for clinical practice.

### 5.5 Strengths and limitations

This overview appraised the effectiveness of Vitamin D supplements in treating KOA on existing literature. We strictly adhered to the principles of dual-reviewer selection, data extraction, and quality evaluation. A third reviewer was consulted to resolve any disputes so that the overview results were as reliable as possible. We used AMSTAR-2, PRISMA, and ROBIS assessment tools to report the reporting quality, methodological quality, and risk of bias of systematic reviews, and used GRADE to evaluate the quality of evidence for clinical outcomes. Lastly, we used the CCA index to illustrate the degree of overlap among different systematic reviews regarding the primary studies. Using citation matrices and heat maps, there was some overlap and consensus among studies on vitamin D supplementation, although differences existed in certain outcome measures. This study differentiated between different control types and included a wider range of outcome measures. However, the article still has certain limitations. For example, the original studies had various study designs, and the evaluation results of SR/MA exhibited high heterogeneity. Furthermore, the included RCTs were all derived from existing systematic reviews and were not retrieved again, which may introduce the risk of missed studies. Therefore, this may impede an overall assessment of this study.

## 6 Conclusion

The overview findings suggest that Vitamin D supplementation shows promise in the treatment of KOA. The evidence indicates that Vitamin D supplements may improve patients’ Total WOMAC scores, as well as synovial fluid volume in the joints. However, it is important to acknowledge the limitations of the original studies, which have a high risk of bias, and the low methodological quality of the systematic reviews, which diminishes the reliability of the results. Despite these challenges, we still recognize the potential value of Vitamin D supplements as a convenient treatment option for KOA. Future research must prioritize the quality of the original studies and the quality of the evidence from systematic reviews. By focusing on these areas, researchers can provide stronger, more scientific evidence about the effectiveness and safety of vitamin D supplements for KOA. This will help to better understand the benefits and limitations of vitamin D supplements and guide healthcare professionals in making informed decisions for people with KOA.

## Data Availability

The original contributions presented in the study are included in the article/Supplementary material, further inquiries can be directed to the corresponding author.
