# Supplementary material for: Shining light on knee osteoarthritis: an overview of vitamin D supplementation studies
Source: Front Med (Lausanne). 2025 Jan 22;11:1423360. doi: 10.3389/fmed.2024.1423360 (PMC11809650; doi:10.3389/fmed.2024.1423360)
Supplement: Supplementary file 1 [file Table_1.DOCX]

| **Appendix1 Table1. Search strategy adopted in the study.** | | | |
| --- | --- | --- | --- |
| Database | Step | Search criteria | Results |
| Pubmed | #1 | "vitamin d"[MeSH Terms] OR "vitamin d"[All Fields] OR "ergocalciferols"[MeSH Terms] OR "ergocalciferols"[All Fields] OR ("ergocalciferols"[MeSH Terms] OR "ergocalciferols"[All Fields] OR ("vitamin"[All Fields] AND "d2"[All Fields]) OR "vitamin d2"[All Fields]) OR ("cholecalciferol"[MeSH Terms] OR "cholecalciferol"[All Fields] OR ("vitamin"[All Fields] AND "d3"[All Fields]) OR "vitamin d3"[All Fields]) OR ("25 hydroxyvitamin d"[Supplementary Concept] OR "25 hydroxyvitamin d"[All Fields] OR "25 hydroxyvitamin d"[All Fields] OR "calcifediol"[MeSH Terms] OR "calcifediol"[All Fields]) OR (("vitamin d"[MeSH Terms] OR "vitamin d"[All Fields] OR "ergocalciferols"[MeSH Terms] OR "ergocalciferols"[All Fields]) AND ("supplemental"[All Fields] OR "supplementating"[All Fields] OR "supplementation"[All Fields] OR "supplementation s"[All Fields] OR "supplementations"[All Fields] OR "supplemention"[All Fields])) OR ("25-hydrox-vitamin"[All Fields] AND "D"[All Fields]) OR ("vitamin d"[MeSH Terms] OR "vitamin d"[All Fields] OR "vit d"[All Fields]) OR "1alpha 25 dihydroxycholecalciferol"[All Fields] OR ("1 25 dihydroxyvitamin d"[Supplementary Concept] OR "1 25 dihydroxyvitamin d"[All Fields] OR "1 25 dihydroxyvitamin d"[All Fields]) OR "25OHD"[All Fields] OR ("calcifediol"[MeSH Terms] OR "calcifediol"[All Fields]) OR ("calcitriol"[MeSH Terms] OR "calcitriol"[All Fields] OR "calcitriols"[All Fields]) OR ("ergocalciferols"[MeSH Terms] OR "ergocalciferols"[All Fields] OR "calciferol"[All Fields] OR "calciferols"[All Fields]) OR ("cholecalciferol"[MeSH Terms] OR "cholecalciferol"[All Fields] OR "cholecalciferols"[All Fields] OR "colecalciferol"[All Fields]) OR ("ergocalciferols"[MeSH Terms] OR "ergocalciferols"[All Fields] OR "ergocalciferol"[All Fields]) OR ("cholecalciferol"[MeSH Terms] OR "cholecalciferol"[All Fields] OR "cholecalciferols"[All Fields] OR "colecalciferol"[All Fields]) OR ("dihydroxycholecalciferols"[MeSH Terms] OR "dihydroxycholecalciferols"[All Fields] OR "dihydroxycholecalciferol"[All Fields]) | 108,922 |
|  | #2 | "osteoarthritis, knee"[MeSH Terms] OR ("osteoarthritis"[All Fields] AND "knee"[All Fields]) OR "knee osteoarthritis"[All Fields] OR ("knee"[All Fields] AND "osteoarthritis"[All Fields]) | 50,191 |
|  | #3 | "systematic review"[Publication Type] OR "systematic reviews as topic"[MeSH Terms] OR "systematic review"[All Fields] OR "systematic review"[Publication Type] OR "systematic reviews as topic"[MeSH Terms] OR "systematic reviews"[All Fields] OR "meta analysis"[Publication Type] OR "meta analysis as topic"[MeSH Terms] OR "meta analysis"[All Fields] OR "meta analysis"[Publication Type] OR "meta analysis as topic"[MeSH Terms] OR "meta analyses"[All Fields] | 482,889 |
|  | #4 | #1 AND #2 AND #3 | 23 |
| Web of science | #1 | TS=(Vitamin D OR Vitamin D2 OR Vitamin D3 OR 25-hydroxyvitamin D OR Vitamin D Supplementation OR 25-hydrox-vitamin D OR Vit D OR 1alpha,25-Dihydroxycholecalciferol OR 1,25-dihydroxyvitamin D OR 25OHD OR Calcifediol OR Calcitriol OR calciferol OR Cholecalciferol OR Ergocalciferol OR Colecalciferol OR Dihydroxycholecalciferol) | 207,924 |
|  | #2 | TS=（Knee Osteoarthritis） | 80,694 |
|  | #3 | TS=（Systematic review OR Systematic reviews OR meta-analysis OR meta-analyses） | 696,179 |
|  | #4 | #1 AND #2 AND #3 | 59 |
| Embase | #1 | vitamin AND d OR (vitamin AND d2) OR (vitamin AND d3) OR ('25 hydroxyvitamin' AND d) OR (vitamin AND d AND supplementation) OR (25 AND hydrox AND vitamin AND d) OR (vit AND d) OR (1alpha,25 AND dihydroxycholecalciferol) OR (1,25 AND dihydroxyvitamin AND d) OR 25ohd OR calcifediol OR calcitriol OR calciferol OR cholecalciferol OR ergocalciferol OR colecalciferol OR dihydroxycholecalciferol | 275,658 |
|  | #2 | （knee AND osteoarthritis） | 79,930 |
|  | #3 | systematic AND review OR (systematic AND reviews) OR 'meta analysis' OR 'meta analyses' | 811,642 |
|  | #4 | #1 AND #2 AND #3 | 77 |
| Cochrane library | #1 | (Vitamin D) OR (Vitamin D2) OR (Vitamin D3) OR (25 hydroxyvitamin D) OR (Vitamin D Supplementation) OR (25 hydrox vitamin D) OR (Vit D) OR (1alpha,25 Dihydroxycholecalciferol) OR (1,25 Dihydroxyvitamin D) OR 25OHD OR Calcifediol OR Calcitriol OR calciferol OR Cholecalciferol OR Ergocalciferol OR Colecalciferol OR Dihydroxycholecalciferol | 22,559 |
|  | #2 | (Knee Osteoarthritis) | 16,812 |
|  | #3 | (Systematic review) OR (Systematic reviews) OR (meta-analysis) OR (meta-analyses) | 47,052 |
|  | #4 | #1 AND #2 AND #3 | 15 |

| **Appendix1 Table2. List of excluded studies, with reasons for exclusion after full text reading.** | |
| --- | --- |
| **References** | **Reason for exclusion** |
| Jin, X. , Antony, B. , Wang, X. , Persson, M. S. , Mcalindon, T. , & Arden, N. K. , et al. (2020). Protocol: effect of vitamin d supplementation on pain and physical function in patients with knee osteoarthritis (oa): an oa trial bank protocol for a systematic review and individual patient data (ipd) meta-analysis. BMJ Open, 10(4). | A protocol |
| Yuelong, C. , Tania, W. , Kay, N. , Jianhao, L. , Graeme, J. , & Changhai, D. . (2013). Association between serum levels of 25-hydroxyvitamin d and osteoarthritis: a systematic review. Rheumatology(7), 1323-1334. | Serum levels of 25-hydroxyvitamin D was the main intervention. |
| Salman, Hussain, Ambrish, Singh, Mohd, & Akhtar, et al. (2017). Letter to the editor on the article "the effect of vitamin d supplementation on knee osteoarthritis: a meta-analysis of randomized controlled trials". International Journal of Surgery. | A letter |
| Zhao-Hua, Z. , Xing-Zhong, J. , Weiya, Z. , Mao, C. , Dong-Qing, Y. , & Yu, Z. , et al. (2014). Associations between vitamin d receptor gene polymorphisms and osteoarthritis: an updated meta-analysis. Rheumatology(6), 998-1008. | Genetic studies |
| Balanescu, A. , Bischoff-Ferrari, H. A. , Souza, S. D. , Dorner, T. E. , Putrik, P. , & Lucía Silva-Fernández, et al. (2022). Effects of diet on the outcomes of rheumatic and musculoskeletal diseases (rmds): systematic review and meta-analyses informing the 2021 eular recommendations for lifestyle improvements in people with rmds. RMD Open, 8(2), 18001-66. | KOA patients were not the main intervention group. |
| Li, C. L. . (2021). Vitamin d receptor gene polymorphisms and osteoarthritis: a meta-analysis. Rheumatology, 60(2). | Genetic studies |
| Liu, H. , He, H. , Li, S. , Yang, L. , Wang, P. , & Liu, C. , et al. (2014). No. 57 vitamin d receptor gene polymorphisms and risk of osteoarthritis: a meta-analysis. Experimental Biology & Medicine, 6(5), S94-S94. | Genetic studies |
| Lee, Y. H. , Woo, J. H. , Choi, S. J. , Ji, J. D. , & Song, G. G. . (2009). Vitamin d receptor taqi, bsmi and apai polymorphisms and osteoarthritis susceptibility: a meta-analysis. Joint Bone Spine, 76( 2), 156-161. | Genetic studies |
| Liu, X. , Machado, G. C. , Eyles, J. P. , Ravi, V. , & Hunter, D. J. . (2017). Dietary supplements for treating osteoarthritis: a systematic review and meta-analysis. British Journal of Sports Medicine, S292-S293. | KOA patients were not the main intervention group. |
| Beaudart, C. , L. Lengelé, Leclercq, V. , Geerinck, A. , & Reginster, J. Y. . (2020). Symptomatic efficacy of pharmacological treatments for knee osteoarthritis: a systematic review and a network meta-analysis with a 6-month time horizon. Drugs, 80(5), 1-13. | A Network Meta‑Analysis |
| Bergink, A. P. , Zillikens, M. C. , Leeuwen, J. P. T. M. V. , Hofman, A. , & Meurs, J. B. J. V. . (2016). 25-hydroxyvitamin d and osteoarthritis: a meta-analysis including new data. Seminars in Arthritis & Rheumatism, 45(5), 539-546. | Serum levels of 25-hydroxyvitamin D was the main intervention. |
| McAlindon, T., & Osani, M. (2019). VITAMIN D AND OSTEOARTHRITIS. OSTEOPOROSIS INTERNATIONAL, 30, S205–S205. | A meeting abstract |
