# Supplementary material for: Shining light on knee osteoarthritis: an overview of vitamin D supplementation studies
Source: Front Med (Lausanne). 2025 Jan 22;11:1423360. doi: 10.3389/fmed.2024.1423360 (PMC11809650; doi:10.3389/fmed.2024.1423360)
Supplement: Supplementary file 2 [file Table_2.DOCX]

**Appendix2. Report quality evaluation included in SRs/MAs by PRISMA 2020.**

| **Section** | **Item** | | **Wang**  **2023** | **Mathieu**  **2022** | **Zhao**  **2021** | **Yu**  **2021** | **Vaishya**  **2019** | **Hussain**  **2017** | **Diao**  **2017** | **Gao**  **2017** | **Gallagher**  **2014** | **Compliance**  **(%)** |
| --- | --- | --- | --- | --- | --- | --- | --- | --- | --- | --- | --- | --- |
| **Title** | 1.Title | | Y | Y | Y | Y | Y | Y | Y | Y | Y | 100 |
| **Abstract** | 2.Abstract | | Y | Y | Y | Y | Y | Y | Y | Y | Y | 100 |
| **Introduction** | 3.Rationale | | Y | Y | Y | Y | PY | Y | Y | Y | PY | 77.8 |
|  | 4.Objectives | | Y | Y | Y | Y | Y | Y | Y | Y | Y | 100 |
| **Methods** | 5.Eligibility criteria | | Y | Y | Y | Y | PY | Y | Y | PY | Y | 88.9 |
|  | 6.Information sources | | PY | Y | PY | PY | PY | Y | Y | PY | Y | 44.4 |
|  | 7.Search strategy | | Y | Y | Y | PY | PY | Y | Y | Y | PY | 66.7 |
|  | 8.Selection process | | Y | Y | N | PY | Y | Y | Y | Y | PY | 66.7 |
|  | 9.Data collection process | | Y | Y | PY | PY | Y | Y | Y | PY | N | 55.6 |
|  | 10a | Data items | Y | Y | Y | Y | Y | Y | Y | Y | Y | 100 |
|  | 10b |  | PY | Y | Y | PY | PY | PY | PY | N | PY | 22.2 |
|  | 11.Study risk of bias  assessment | | Y | Y | Y | N | N | Y | Y | Y | Y | 77.8 |
|  | 12.Effect measures | | Y | Y | Y | Y | N | N | Y | Y | N | 66.7 |
|  | 13a | Synthesis methods | Y | Y | Y | Y | Y | Y | Y | Y | Y | 100 |
|  | 13b |  | N | PY | PY | N | N | N | PY | N | N | 0 |
|  | 13c |  | Y | Y | Y | Y | N | Y | Y | Y | N | 77.8 |
|  | 13d |  | Y | Y | Y | Y | N | Y | Y | Y | N | 77.8 |
|  | 13e |  | Y | Y | Y | Y | N | Y | Y | N | N | 66.7 |
|  | 13f |  | N | N | Y | Y | N | N | Y | N | N | 33.3 |
|  | 14 | Reporting bias assessment | N | N | Y | Y | N | N | Y | N | N | 33.3 |
|  | 15.Certainty assessment | | N | N | N | N | N | N | N | Y | N | 11.1 |
| **Results** | 16a | Study selection | Y | Y | Y | Y | Y | Y | Y | Y | Y | 100 |
|  | 16b |  | Y | Y | Y | Y | Y | Y | Y | Y | Y | 100 |
|  | 17.Study characteristics | | Y | Y | Y | Y | Y | Y | Y | Y | Y | 100 |
|  | 18.Risk of bias in studies | | N | N | Y | Y | N | N | Y | N | N | 33.3 |
|  | 19.Results of individual studies | | Y | Y | Y | Y | N | Y | Y | Y | Y | 88.9 |
|  | 20a | Results of syntheses | N | N | N | N | N | N | N | N | N | 0 |
|  | 20b |  | Y | Y | Y | Y | Y | Y | Y | Y | N | 88.9 |
|  | 20c |  | N | N | N | Y | N | N | Y | N | N | 22.2 |
|  | 20d |  | N | N | Y | Y | N | N | Y | N | N | 33.3 |
|  | 21.Reporting biases | | N | N | Y | Y | N | N | Y | N | N | 0 |
|  | 22.Certainty of evidence | | N | N | N | N | V | N | N | Y | N | 11.1 |
| **Discussion** | 23a | Discussion | Y | Y | Y | Y | Y | Y | Y | Y | Y | 100 |
|  | 23b |  | PY | Y | N | Y | PY | PY | N | N | N | 22.2 |
|  | 23c |  | Y | Y | Y | Y | Y | Y | Y | Y | Y | 100 |
|  | 23d |  | Y | Y | PY | Y | PY | PY | PY | PY | Y | 44.4 |
| **Other Information** | 24a | Registration and protocol | Y | Y | N | N | N | Y | N | N | N | 33.3 |
|  | 24b |  | Y | Y | N | N | N | Y | N | N | N | 33.3 |
|  | 24c |  | Y | Y | N | N | N | Y | N | N | N | 33.3 |
|  | 25.Support | | Y | Y | Y | Y | Y | Y | N | Y | Y | 88.9 |
|  | 26.Competing interests | | Y | Y | Y | Y | Y | N | Y | Y | Y | 88.9 |
|  | 27.Availability of data,  code and other materials | | Y | Y | N | N | N | Y | N | N | Y | 44.4 |

**Y**:Yes **PY**:Partial Yes  **N**:No

**Item 1**: Identify whether this report is a systematic review; **Item 2**: Check the PRISMA 2020 Abstracts checklist; **Item 3**: Describe the rationale for this systematic review; **Item 4**: Articulate a clear statement of the objectives or questions addressed by the review; **Item 5**: Specify the inclusion and exclusion criteria for the review; **Item 6**: Specify all sources used for retrieval or inquiry, and indicate the last search or inquiry date for each source. Refine for conciseness and clarity; **Item 7**: Provide comprehensive search strategies; **Item 8**: retrieval and screening process; **Item 9**: Data collection process; **Item 10a**: List and define all outcomes for which data were sought; **Item 10b**: List and define all other variables for which data were sought; **Item 11**: Study risk of bias assessment; **Item 12**: Effect measures; **Item 13a**: Enumerate each study analysis process and intervention characteristic; **Item 13b**: Describe any methods required to prepare the data for presentation or synthesis; **Item 13c**: Describe any methods employed to tabulate or visually present the results of individual studies and syntheses; **Item 13d**: Describe any methods used to synthesize results; **Item 13e**: Describe any methods used to explore possible causes of heterogeneity among study results; **Item 13f**: Describe any sensitivity analyses conducted to assess robustness of the synthesized results; **Item 14**: Reporting bias assessment; **Item 15**: Certainty assessment; **Item 16a**: search and selection process; **Item 16b**: Cite studies that may seem to meet the inclusion criteria but were excluded, and provide an explanation for their exclusion; **Item 17**: Study characteristics; **Item 18**: Risk of bias in studies; **Item 19**: Results of individual studies; **Item 20a**: Briefly summarize the characteristics and risk of bias among participating studies; **Item 20b**: Present results of all statistical syntheses conducted; **Item 20c**: Present findings from all inquiries into potential causes of heterogeneity among study results; **Item 20d**: Present findings from all sensitivity analyses performed to evaluate the robustness of the synthesized results; **Item 21**: Reporting biases; **Item 22**: Certainty of evidence; **Item 23a**: Provide a general interpretation of the results in the context of other evidence; **Item 23b**: Discuss any limitations of the evidence included in the review; **Item 23c**: Discuss any limitations of the review processes used; **Item 23d**: Discuss implications of the results for practice, policy, and future research; **Item 24a** Provide registration details for the review, including the register name and registration number, or state if the review was not registered; **Item 24b**: Specify the location where the review protocol can be accessed or state that a protocol was not prepared; **Item 24c**: Describe and clarify any changes made to the information provided during registration or outlined in the protocol; **Item 25**: Support; **Item 26**: Competing interests; **Item 27**: Availability of data, code and other materials
